# Shifts in survival and reproduction after chronic warming enhance the potential of a marine copepod to persist under extreme heat events

**DOI:** 10.1093/plankt/fbad037

**Published:** 2023-09-06

**Authors:** Carlos de Juan, Albert Calbet, Enric Saiz

**Affiliations:** Department of Marine Biology and Oceanography, Institut de Ciències del Mar (ICM), CSIC Pg. Marítim de la Barceloneta 37–49, 08003 Barcelona, Spain; Department of Marine Biology and Oceanography, Institut de Ciències del Mar (ICM), CSIC Pg. Marítim de la Barceloneta 37–49, 08003 Barcelona, Spain; Department of Marine Biology and Oceanography, Institut de Ciències del Mar (ICM), CSIC Pg. Marítim de la Barceloneta 37–49, 08003 Barcelona, Spain

**Keywords:** copepods, temperature, heatwave, tolerance, reproduction, thermal limits

## Abstract

The study of a species’ thermal tolerance and vital rates responses provides useful metrics to characterize its vulnerability to ocean warming. Under prolonged thermal stress, plastic and adaptive processes can adjust the physiology of organisms. Yet it is uncertain whether the species can expand their upper thermal limits to cope with rapid and extreme changes in environmental temperature. In this study, we reared the marine copepod *Paracartia grani* at control (19°C) and warmer conditions (25°C) for >18 generations and assessed their survival and fecundity under short-term exposure to a range of temperatures (11–34°C). After multigenerational warming, the upper tolerance to acute exposure (24 h) increased by 1–1.3°C, although this enhancement decreased to 0.3–0.8°C after longer thermal stress (7 days). Warm-reared copepods were smaller and produced significantly fewer offspring at the optimum temperature. No shift in the thermal breadth of the reproductive response was observed. Yet the fecundity rates of the warm-reared copepods in the upper thermal range were up to 21-fold higher than the control. Our results show that chronic warming improved tolerance to stress temperatures and fecundity of *P. grani*, therefore, enhancing its chances to persist under extreme heat events.

## INTRODUCTION

Marine organisms are currently facing the combined effects of a gradual increase in the mean ocean heat content and an escalation in the frequency, duration and intensity of extreme thermal events (Smith *et al*., 2023). Consequences of such phenomena are already being observed globally, with studies reporting severe reductions in species abundance (Garrabou *et al*., 2022; Edgar *et al*., 2023), phenological (Richardson, 2008), and distributional shifts (Evans *et al*., 2020) and re-organization of species assemblages (Benedetti *et al*., 2021) that result in profound and lasting alterations in marine ecosystems (Suryan *et al*., 2021; Batten *et al*., 2022). In this context, studying the thermal tolerance and temperature effects on the vital rates of species can help assess their responses to changing ocean temperatures and predict ecological trends (Pörtner and Farrell, 2008; Huey *et al*., 2012).

Among marine organisms, copepods represent one of the most abundant and diverse groups, inhabiting almost all marine environments (Ibarbalz *et al*., 2019). Given their pivotal role in marine food webs and global biogeochemical cycles, they are particularly compelling subjects for investigation in the face of rising ocean temperatures (Steinberg and Landry, 2017). Global patterns in the heat tolerance of this group are primarily influenced by specific habitats and annual maximum temperatures (Sasaki and Dam, 2021b). However, changes in habitat temperatures over time can lead to alterations in organismal heat tolerance. Copepods may exhibit a degree of plasticity in their thermal tolerance, allowing them to acclimate to rapid changes in ambient temperature (Jiang *et al*., 2008), similar to other groups of ectotherms (Gunderson *et al*., 2017; Morley *et al.*, 2019). However, prolonged duration of stress strongly affects survival (Jiang *et al*., 2008; Rezende *et al*., 2014), although this factor is often overlooked when assessing species’ heat tolerance. Long-term warming may result in an increase in heat tolerance through the action of intra- and intergenerational responses (Geerts *et al*., 2015). Nonetheless, this enhancement could be constrained by physiological limits (Kelly *et al*., 2012; Morgan *et al*., 2020). Furthermore, following an enhancement in heat tolerance the acclimation capacity to future extreme temperatures could be reduced (Sasaki and Dam 2021a; van Heerwaarden and Kellermann, 2020). In this regard, there is a scarcity of studies that experimentally address changes in heat tolerance through multiple generational exposure in marine zooplankton.

The range of temperatures within which vital processes, such as reproduction, can take place is often narrower than the range required for survival (Halsband-Lenk *et al*., 2002; Feng and Papes, 2017). Consequently, at sub-lethal temperatures, adverse effects on fecundity can impose stricter limits on local persistence, resulting in a decline in population size. Therefore, considering both survival and reproductive responses can offer valuable insights into an organism’s capacity to withstand changing ocean temperatures.

Life-history characteristics, such as reproductive rates, typically show unimodal responses (Saiz *et al*., 2022), where rates increase exponentially as the temperature rises until reaching an optimum temperature (T_opt_). At this T_opt_, rates are at maximum (R_max_), and then they decline rapidly until the loss of the function occurs (CT_max_) (Angilletta *et al*., 2002). Under prolonged thermal stress, adjustments in the physiology of organisms can lower the R_max_ by reducing the thermal sensitivity of metabolic processes or displace the T_opt_ and CT_max_ along the thermal gradient (Schulte *et al*., 2011; Seebacher *et al*., 2015). Predictably, these adaptive responses in vital rates and thermal tolerance are expected to emerge from changes at the phenotypic and genotypic level and play a fundamental role in the thermal response of organisms to habitat conditions, especially for those that, as copepods, show fast development and short generation times (Vehmaa *et al*., 2012; Dam, 2013; Dam *et al*., 2021). However, it is uncertain if both limits can be expanded to an extent that allow these organisms to persist under changing ocean temperatures.

In this study, we compared the thermal tolerance and reproduction response to short-term thermal stress (15–34°C) of a parental population of the marine copepod *Paracartia grani*, which had been cultured in the laboratory under stable conditions (19°C) for over a decade, with a descendent line of *P. grani* reared at a warmer temperature (25°C) for ˃18 generations. *P. grani* is a calanoid copepod member of the Acartidae family, a taxonomic group found in aquatic habitats worldwide (Belmonte, 2021). This species is commonly found in coastal and semi-enclosed waters of the North and South East Atlantic, as well as in the Mediterranean Sea (Rodríguez and Vives, 1984; Razouls *et al*., 2005-2023; Boyer *et al*., 2012). In the western Mediterranean, this species can be found year-round over a wide thermal range (8.3–23.8°C), with peak occurrences between spring and autumn (Rodríguez and Jiménez (1990).

In laboratory conditions, this species has been shown to tolerate an even broader range of temperatures, from 5.7 to 32°C (Saiz *et al*., 2022), and exhibit a high capacity to acclimate to thermal stress (Saiz *et al*., 2022; de Juan *et al*., 2023). The main questions addressed in our study were whether survival and reproduction are enhanced by multigenerational rearing under warming conditions (25°C), and to what extent these shifts can provide an advantage for this species in case of extreme heat events. We hypothesized that multigenerational thermal exposure to warmer temperatures would enhance the thermal tolerance of this species and also improve both the T_opt_ and CT_max_ in its reproductive response. Additionally, building upon previous research (de Juan *et al*., 2023), we investigated whether the magnitude of the effects of a rapid change in temperature on physiological processes would be diminished, resulting in lower Q_10_ values.

## METHOD

### Species rearing conditions

*Paracartia grani* (Sars, 1904) specimens were originally collected in coastal waters north of Barcelona (NW Mediterranean) in 2007–2008 and maintained at 19°C (± 1°C) with 10: 14 light/cycle at the Institut de Ciències del Mar (ICM, CSIC) for > 14 years. Copepods were reared in 20-40 L tanks and routinely fed *ad libitum* three times a week with the cryptophyte *Rhodomonas salina* (strain K-0294, Scandinavian Culture Collection of Algae and Protozoa), grown in f/2 medium. From this culture, two separated lines were established to be used for the experiments, one at 19°C (“Control”) and another one at 25°C (“Warm”). The temperature was maintained stable (± 0.1°C) using two 150 L water baths with TECO chiller and heater units, and the light regime was set to 15–20 μE m^−2^ s^−1^ and 10: 14 h light/dark cycle. We monitored the development of the cohorts and, according to life stage and copepod density, we adjusted the volume of *R. salina* added to keep copepods in satiation or close to satiation conditions (from 2 ppm for early stage nauplii to 10 ppm for adults; Olivares *et al*., 2019). Once the cultures were dominated by mature adults, freshly spawned eggs were collected by siphoning the bottom of the tank and transferred in batches of 10 000–20 000 into the new 20 L tanks with temperature-acclimated filtered (0.1 μm) seawater. The subsequent generations were then reared separately to ensure the conditioning of the lines to temperature. Experiments were carried out after being reared for >10 months (>18 generations).

### Experimental setup

The survival and performance of adult female *P. grani* reared under control (19°C) and warm conditions (25°C) were examined by exposing individuals to 11, 15, 19, 22, 25, 27, 29, 30, 31, 31.5, 32, 33 and 34°C (± 0.1°C) during 7 days. To achieve this, we conducted the following procedure: once the majority of individuals in the cohorts had reached the adult stage (both males and females), we ensured their nourishment by feeding them a satiating diet of *R. salina* for a period of 2–5 days to guarantee fertilization. Subsequently, we separated adults from the culture with a 250-μm sieve, and we transferred groups of 20 females into 1 L Pyrex bottles using a wide-mouth Pasteur pipette. These bottles were filled with a saturating suspension of *R. salina* (9 ppm, 1 144 μg C L^−1^), which had been previously acclimated to the respective temperatures. To ensure that nutrient availability was not limited at higher temperatures, we supplemented the *R. salina* suspensions with f/2 medium, aiming to achieve a nutrient load equivalent to ~f/10 medium in the final suspension. The concentration of prey and copepod density in the bottles were calculated based on previous knowledge to ensure food-saturating conditions during incubation (Olivares *et al*., 2019). The food concentration in the bottles was measured initially and during the experiment using a Beckman Coulter Multisizer III particle counter (aperture tube 100 μm). The experimental bottles were immersed in the water baths and regularly homogenized (three times a day) by gently turning the bottles upside down several times. We ran at least triplicate bottles for each test temperature and rearing condition. In total, 1800 (900 control +900 warm) individuals were used in the assays to assess the tolerance and reproductive thermal responses. After 24 h, we filtered the content of the bottles through 100 μm and 20 μm sieves; adults, retained by the 100-μm mesh, were inspected and counted under the stereo-microscope and their survival was checked. Copepods were considered dead if they did not swim or react to physical stimulation. Alive individuals were returned to the same suspensions using a Pasteur pipette. The fraction between 100 and 20 μm, containing eggs, nauplii and fecal pellets, was discarded. We renewed the food suspensions in the bottles every 48 h by gently siphoning the old suspension using a pipette tip fitted with 100-μm mesh (reverse filtration) and then refilled the incubation bottles with the new temperature-conditioned suspension of *R. salina*. After 6 days, the bottle content was filtered through 100-μm and 20-μm sieves to separate, respectively, adults and eggs from the suspension and to check the survivorship as previously described. We refilled the bottles with fresh suspensions of *R. salina* and returned the adult females to the bottles to assess egg production rates. The following day, we repeated the procedure described before and ended the experiment. We checked the final survival of the copepods in the bottles and preserved the remaining alive individuals in 4% formaldehyde. The fraction containing the eggs and pellets (20-μm mesh) was fixed in a 2% Lugol solution. Posteriorly, we counted the eggs, egg shells and hatched nauplii using an inverted microscope (Nikon Diaphot 200) and calculated egg production rates as the total number of eggs laid divided by the number of females in the incubation and the incubation time. To determine the effect of ambient temperature on egg diameter, for each test temperature and rearing condition we took pictures of 30 preserved eggs (all from a single replicate) under an inverted microscope. Additionally, we took pictures of the adults at each rearing condition initially and the preserved adults after the stress period (only in the upper thermal range; *n* = 30 for each test temperature and rearing condition) to check whether there was a relationship between the size of the female and the survival at the lethal temperatures. All pictures were processed using ImageJ software (v1.53v). The size of the adult was measured as the length of the prosome, which is the linear distance from the upper end of the cephalosome to the last somite of the metasome. The diameter of the egg was determined by adjusting an ellipse to the copepod egg and averaging the *x* and *y* axes.

### Models and calculations

To assess the survival (*S*) of *P. grani* along the thermal gradient, we adjusted a sigmoidal curve following Tarapacki *et al*. (2021):


(1) \begin{equation*} S=\frac{c}{1+\exp \left(T-b\right)} \end{equation*}

where *a* is the slope of the descending phase, *T* is the test temperature (°C), and *b* is the temperature at which 50% mortality is reached in relation to *c*, which is the maximum survival. The temperature causing 50% (LT_50_) and 90% (LT_90_) mortality was then extracted from the fitted curves.

Thermal performance curves of the egg production rates were modeled using the R package *rTPC* (Padfield *et al*., 2021). Based on the global score of the Akaike information criterion (AIC) among nine fitted models (Padfield *et al*., 2021), we chose the empirical model by Rosso *et al*. (1993) to be fitted to the egg production data:

where *R* is the rate of copepod egg production (eggs ind^−1^ d^−1^), *T* is the test temperature (°C), *T_min_* (°C) is the temperature at which the egg production rate is zero, *T_max_* (°C) is the temperature at which the egg production rate is zero, and *T_opt_* (°C) is the optimum temperature at which the maximum egg production rate, *R_max_* (eggs ind^−1^ d^−1^), occurs.

Parameter estimates and 95% confidence intervals were obtained using case resampling bootstrapping (R package *car*, v3.1-1; Fox and Weisberg, 2019). *CT_max_* values represent the estimated rate closest to 0 at the upper limit and it is equivalent to the model *T_max_*. Additionally, we also assessed the *thermal breadth*, defined as the range of temperatures where performance is 80% of the maximum rate, and the *thermal safety margin,* calculated as the difference between *T_opt_* and the rearing temperature. We calculated the *Q_10_* coefficients for egg production, i.e. the fold variation in a given rate for a 10°C increase, from the slopes of the linear regression between the natural logarithm of the estimated egg production rates and temperature, as *exp*^(10 x *slope)*^.

As a proxy for fitness, we multiplied the adult survival rate and the offspring produced (egg production rates), both taken from the fitted curves after 7 days of exposure, to determine the potential recruitment of both control and treatment at all test temperatures.

### Data analysis

Estimates of the curve parameters were compared by examining the 95% confidence intervals and considered to differ significantly if they did not overlap. The differences in female prosome length under both rearing conditions were analyzed using a two-tailed Student’s test. Additionally, two-way ANOVA and Dunnett post hoc comparisons were conducted to assess whether the body size of females that survived at higher temperatures (27°C and 32°C) differed from their respective controls.

As egg diameter and temperature followed an exponential relationship, a comparison between the intercepts and slopes of the control and treatment was carried out using ANCOVA analysis on ln-transformed egg diameter. All plots and statistical analyses were performed in R software (v4.1.1; R Core Team, 2021).


(2) \begin{equation*} R=\frac{R_{\mathrm{m}\mathrm{ax}}\times{\left(\left(T-{T}_{\mathrm{m}\mathrm{ax}}\right)\left(T-{T}_{\mathrm{m}\mathrm{in}}\right)\right)}^2}{\left({T}_{opt}-{T}_{\mathrm{m}\mathrm{in}}\right)\left(\left({T}_{opt}-{T}_{\mathrm{m}\mathrm{in}}\right)\left(T-{T}_{opt}\right)-\left({T}_{opt}-{T}_{\mathrm{m} ax}\right)\left({T}_{opt}+{T}_{\mathrm{m}\mathrm{in}}-2T\right)\right)} \end{equation*}

## RESULTS

### Heat tolerance

**Fig. 1:**
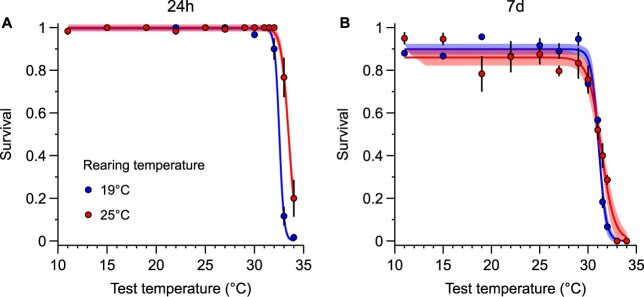
Survival curves for adult females of *P. grani* reared under control (19°C) and warm (25°C) conditions after 24 h **(A)** and 7 days **(B)** of exposure to a range of test temperatures. Each point represents the average of 3–6 replicate bottles for each test temperature, and the error bars show the standard errors. The lines and shades show the fitted sigmoidal curves and the corresponding 95% confidence intervals, respectively.

After 24 h of exposure, survival was high, close to 100%, for the control (19°C) and warm-reared (25°C) treatments across the thermal range comprised between 11°C and 31.5°C (Fig. 1A). From 32°C, survival of the control population started to decrease, whereas in the warm-reared copepods, this drop appeared after 33°C. In the descending phase of the curve, survival was consistently higher for warm-reared copepods. Consequently, LT_50_ was 32.5°C (95% CI 32.47–32.59) for the control and 33.5°C (95% CI 33.37–33.56) for the warm-reared treatment. The LT_90_ after 24 h was 33.0°C (95% CI: 32.98–33.10) and 34.3°C (95% CI: 34.20–34.39) for the control and warm-reared treatments, respectively.

After 7 days, survival for both populations was still high, between 11 and 30°C with a maximum survival of 85% (Fig. 1B). However, the upper thermal tolerance was significantly reduced in the control and warm-reared treatments, and the differences between them smoothed, with LT_50_ values of 31.1°C (95% CI: 30.95–31.21) and 31.4°C (95% CI: 31.13–31.59), respectively. At the most extreme temperatures (31.5 and 32°C), however, the survival of warm-reared copepods was still 2.2 and 5.4 times higher, respectively, than that of the control treatment. LT_90_ also decreased in both treatments but remained significantly higher in warm-reared copepods (control: 31.9°C [95% CI: 31.68–32.11]; warm-reared: 32.7°C [95% CI: 32.28–33.15]).

### Body size

**Fig. 2:**
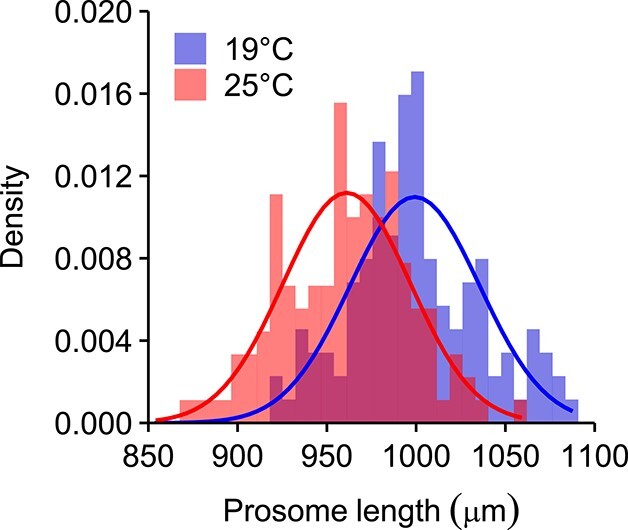
Density plot for prosome length of adult females of *P. grani* reared in control (19°C) and warm (25°C) conditions. Lines show Gaussian fit.

Individuals reared at 19°C were generally larger than those reared at 25°C (*P* < 0.001), averaging 999.3 (± 3.3SE) and 961.1 (± 3.2SE) μm, respectively (Fig. 2). The homogeneity test did not show differences in body size variance between control and warm-reared treatments (F_121, 124_ = 1.04, *P* = 0.841), with similar coefficients of variation (control: 3.6%; warm-reared: 3.5%). The body length of adults who survived extreme temperatures (27–32°C) did not differ from that of their respective rearing temperature, neither for 19°C (F_1,216_ = 0.528, *P* = 0.468) nor for 25°C (F_1, 213_ = 0.133, *P* = 0.716).

### Egg production rate

**Fig. 3:**
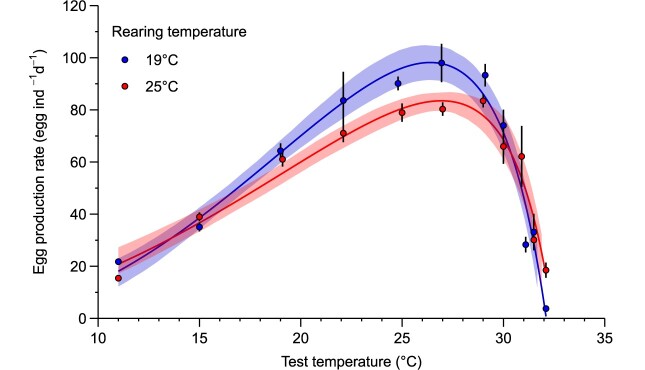
Thermal performance curves of the egg production rate of female *P. grani* reared under control (19°C) and warm (25°C) conditions after 7 days of exposure. Each point shows averages (*n* = 3–6 for each test temperature), and error bars show standard errors. Lines show the model fit (see Methods) and shades show the 95% confidence intervals estimated using case resampling.

Surviving females produced offspring at all temperatures tested (Fig. 3). The maximum observed egg production rate for the copepods reared at 19°C was 98 ± 7.4 eggs ind^−1^ d^−1^ when exposed to 27°C, while for the 25°C reared copepods the maximum recorded value was 83.4 ± 2.6 eggs ind^−1^ d^−1^ at 29°C. Table I shows the estimated parameters of the fitted thermal performance curve. The R_max_ of the warm-reared copepods was significantly lower than the control. The T_opt_ was not significantly modified by multigenerational rearing, but the amplitude of the confidence intervals was larger under warm conditions (larger optimum plateau). CT_max_ was not significantly different from the control either, although the values found in the descending phase of the response curve of warm-reared copepods were up to 21 times higher than the control. Consequently, there was no significant difference in thermal breadth between the two rearing conditions. However, the thermal safety margin decreased after multigenerational warming, primarily due to the preservation of T_opt_.

**Table I:** Parameter values of the fitted thermal performance curve of the P. grani egg production rate reared at control (19°C) and warm (25°C) conditions. Otherwise indicated, all units are °C

	19°C			25°C		
Parameter	Estimate	95% CI		Estimate	95% CI	
		Lower limit	Upper limit		Lower limit	Upper limit
R_max_ (eggs ind^−1^ d^−1^)	98.2	91.1	106.5	83.5	78.8	86.6
T_opt_	26.4	25.9	27.2	26.9	25.9	28.0
CT_max_	32.1	31.9	32.4	32.4	32.3	33.0
Thermal safety margin	7.4	6.9	8.2	1.9	0.9	3
Thermal breadth	8.3	7.8	8.7	8.7	8.2	9.4

The Q_10_ coefficients for the curves fitted to the thermal response of the warm-reared copepods were consistently lower than those of the control treatment (Table II). When considering the direction of the response, the higher Q_10_ coefficients were observed at the cooling phase at both temperatures.

**Table II:** Q_10_ coefficients for the ascending phase of the thermal windows of female P. grani reared at 19°C and 25°C. “Rearing T” refers to the rate registered at the test temperature equal to the respective rearing temperature. “Min. rate” refers to the rate at 11°C and “Max. rate” refers to the rate at the T_opt_ for each curve

Curve phase	19°C	25°C
Global Q_10_ (Min. rate – Max. rate)	2.9	2.4
Cooling Q_10_ (Min. rate – Rearing T)	4.7	2.6
Warming Q_10_ (Rearing T – Max. rate)	1.8	1.2

**Fig. 4:**
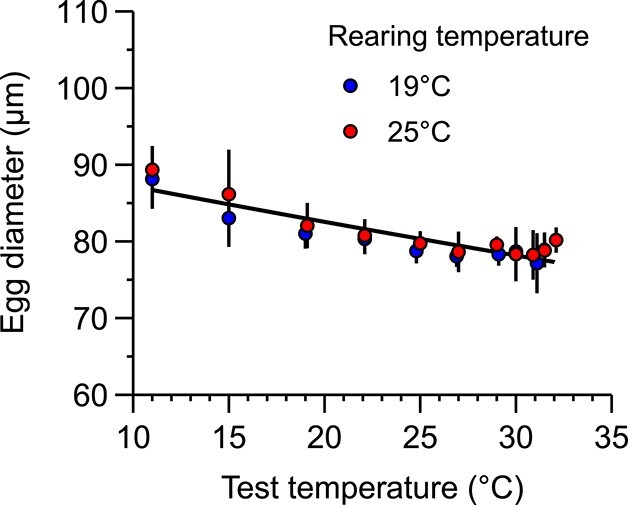
Egg diameter against exposure temperature under control (19°C) and warm conditions (25°C). Individual points show the mean and standard deviation. Lines show the fitted negative exponential.

The diameter of the egg showed a negative trend with the test temperature (Fig. 4) and no differences were found due to the rearing temperature (linear regression of the ln-transformed data; F_1,18_ = 3.38; *P* = 0.08).

### Fitness proxy

**Fig. 5:**
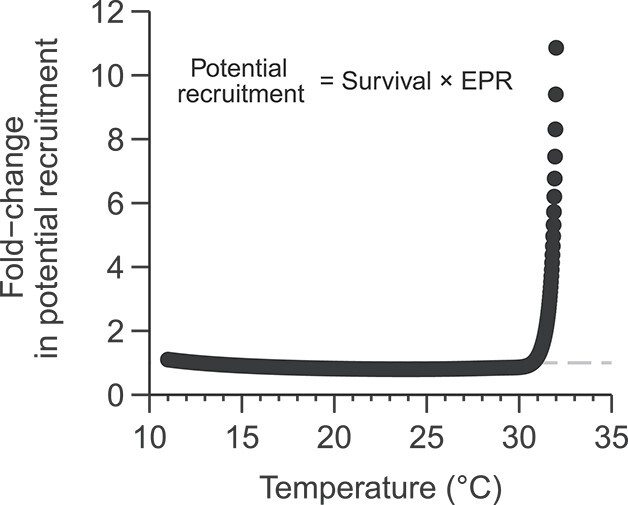
Fold-change in potential recruitment (estimated as the product of the survival and offspring production of females, based on the fitted curves in Figs 1b and 3) of *P. grani* reared under warm conditions (25°C) relative to the control (19°C) after 7 days of exposure to a range of temperatures. The dashed gray line shows a fold-change of 1.0.

The joint effects of temperature on survival and fecundity were analyzed by comparing the potential recruitment index for the control and warm-reared copepods (Fig. 5). At most of the non-stress test temperatures (19–29°C), at which mortality at the control and warm-reared treatments was negligible, the higher fecundity of individuals reared at 19°C determined a higher recruitment potential. In the upper temperature range (30–32°C), at which substantial mortality occurs, the individuals reared at 25°C achieved higher survival and also produced higher fecundity shifting the trend.

## DISCUSSION

### Thermal tolerance

We have shown that the temperate copepod *P. grani* reared in warmer conditions (+6°C) for > 18 generations increased the LT_50_ and LT_90_ in acute exposure (24 h) by 1 and 1.3°C, respectively. However, this enhancement decreased to 0.3 and 0.8°C after longer exposure (7 days). From these results arise that both the duration of the exposure and the choice of metric used to measure thermal tolerance can significantly affect the outcomes obtained (Fig. S1). Although the time of exposure to stress conditions is a key factor in determining the thermal tolerance of an organism, it is often overlooked (Terblanche *et al*., 2007; Rezende *et al*., 2014), which can result in significant uncertainty when conducting global comparisons of the vulnerability of ectotherms to extreme heat events (Gunderson and Stillman, 2015; Morley *et al.*, 2019; Weaving *et al*., 2022). As such events, potentially exceeding the thermal tolerance limits, can persist for extended periods (Garrabou *et al*., 2022) and are expected to be more frequent and severe in the coming future (Oliver *et al*., 2021), it becomes more relevant to assess the organism’s response to sustained thermal stress.

The available information on ectotherms (e.g. Gunderson and Stillman, 2015; Morley *et al.*, 2019; Weaving *et al*., 2022) indicates a general limitation in the plasticity of heat tolerance, with the increase in tolerance per 1°C in short-term acclimation temperatures being much lower than 1°C. However, in contrast to acute exposures, the thermal response after multiple generations may involve mechanisms at phenotypic and genotypic level (Sasaki and Dam, 2019; Dam *et al*., 2021; Brennan *et al*., 2022) that could potentially lead to adaptive changes in heat tolerance. Nevertheless, the increase in heat tolerance of *P. grani* we observed in our study (from 0.05 up to 0.22°C per degree increase in the rearing temperatures), in which the copepod populations were conditioned to warmer sub-lethal temperatures over 18 generations, was, in fact, similar to or lower than those reported in the aforementioned acute-response studies. Similarly, Sasaki and Dam (2021a) also found, after rearing 40 and 80 generations the related copepod *Acartia tonsa* at +4°C conditions, comparable increases in acute heat tolerance (ranging 0.1–0.5°C). A stronger selective pressure than that used in our experiments during rearing (25°C) could favor the selection of the most heat-tolerant genotypes and thereby amplify the tolerance response. However, studies on experimental evolution report the presence of hard physiological limits to the increase in heat tolerance (Morgan *et al*., 2020). For instance, in the intertidal copepod *Tigriopus californicus*, a strong selection for tolerant phenotypes over 5 and 10 generations resulted in only a 0.5°C increase in heat tolerance, showing significant latitudinal differences (Kelly *et al*., 2012). Interestingly, in our experiments, the reduced survival capacity of the warm-reared copepods at extreme temperatures after a longer exposure could suggest an increase in their plasticity rather than a mean increase in their basal heat tolerance (Fig. S1) (van Heerwaarden and Kellermann, 2020).

### Reproductive response

At sub-lethal temperatures, the direct effects of temperature on other life-history traits (i.e. growth and reproduction) can set narrower limits for the persistence of the species. It has long been recognized that energy investments in survival can be at the cost of reproductive efforts and vice versa (Stearns, 1989; Truong *et al*., 2022). In our study, *P. grani* reared under warm conditions could expand both survival and reproduction at extreme temperatures, providing further evidence of a strong coupling between both traits in this species under thermal stress (Saiz *et al*., 2015). Contrary to our hypothesis, we did not observe significant shifts in the T_opt_ of the reproductive response of *P. grani* following multigenerational warming, resulting in a decrease in the thermal safety margin; the thermal breadth of the reproductive response, however, was not altered by the rearing temperature. Moreover, the warm-reared copepods exhibited higher fecundity rates (up to 21-fold) at the thermal extremes despite their smaller size, revealing a shift in the limits of the reproductive response. The observed decrease in R_max_ of the warm-reared copepods can be attributed not only to their smaller size (Ban, 1994; Halsband-Lenk *et al*., 2002) but also to the action of physiological compensation processes (Saiz *et al*., 2022; de Juan *et al*., 2023).

The decrease in the Q_10_ coefficients of the warm-reared copepods provides additional evidence of a reduction in the thermal sensitivity of the female reproductive rate (de Juan *et al*., 2023). Depending on the phase of the curve considered in relation to the rearing temperature, the Q_10_ coefficients varied, suggesting a change in the shape of the curve (i.e. hysteresis) and emphasizing the importance of considering the interaction between previously experimented temperature and the direction of the thermal change (Sinclair *et al*., 2016). However, at lethal temperatures (30–33°C), the warm-reared copepods showed higher survival and produced more offspring than the control treatment (Fig. S2). Consequently, the improvement of survival and reproduction increased the potential recruitment of warm-reared copepods after 7 days at thermal extremes. Therefore, small shifts at the physiological level can scale up to the population level and have ecological consequences (Pörtner and Farrell, 2008).

### Temperature and body size

Temperature also has well-recognized effects on body size (Forster and Hirst, 2012) and its occurrence complicates the evaluation of the thermal responses of ectotherms (Riemer *et al*., 2018). As a major trait that governs all energy and nutrient fluxes, body size effects overlap with thermal effects on physiology (de Juan *et al*., 2023). Examples of thermally driven changes in size and distribution have been observed in marine ectotherms, including marine zooplankton (Evans *et al.*, 2020). Shifts in biodiversity and abundance of this key group may affect its direct links in the trophic chain (Brosset *et al*., 2016) and, consequently, may ultimately alter the functioning of marine food webs and the carbon sequestration capacity for the oceans. Here, the reduction in adult body size at warmer temperatures had scaling effects on egg production, resulting in lower potential recruitment. Recent studies also suggest that animals with smaller body sizes could benefit from an increase in heat tolerance and, at the same time, exhibit lower endurance capacity to longer exposures (Peralta-Maraver and Rezende, 2021). However, we did not observe any significant differences between the body sizes of the females that survived the extreme temperatures that could support the occurrence of such phenomena. In addition, given the relatively small difference in adult size between the warm-reared and control populations (0.6% °C^−1^), any benefits on the tolerance after prolonged exposition to lethal temperatures would possibly be undetectable. Likely, larger size differences, like those driven by interspecific or ontogenetic comparisons, would be required to discern the effect on tolerance. Female size did not affect egg size, which was found to be highly dependent on immediate ambient temperature, as suggested in previous studies (McLaren *et al*., 1969). This reduction of egg size driven by temperature may have important implications for copepod populations as it has been shown that it can influence egg viability, naupliar survival under starvation (Guisande and Harris, 1995) and ontogenetic development (McLaren *et al*., 1969).

### Scaling our results to natural communities

Many copepod species experience important changes in water temperature throughout their seasonal presence (e.g. Horne *et al*., 2016). Therefore, we could expect an advantage for copepods reared in warmer waters over thermal extremes across population development since these heat spikes primarily occur during the warm seasons. An implication of this is that the plasticity of wild animals subjected to larger environmental fluctuations might be underestimated when using laboratory specimens (Morgan *et al*., 2022). *P. grani* is tolerant to a wide range of temperatures (5.7–32°C, Saiz *et al*., 2022). Before the experiments, this species had been reared for > 14 years under steady thermal conditions (19 ± 1°C), yet it still exhibited notable physiological plasticity and acclimation capacity (Saiz *et al*., 2022; de Juan *et al*., 2023). It was rather unexpected to find that this strong phenotypic plasticity, which in nature would help to cope with the intrinsic environmental variability of their habitat (coastal, estuaries and semi-enclosed waters), could be maintained after culturing in the laboratory for so long under stable conditions. Contrarily, other copepod life-history traits, such as feeding and egg production diel rhythms, can be less fixed and quickly affected after laboratory culturing (Tiselius *et al*., 1995; Olivares *et al*., 2020).

In nature, copepods employ various strategies to cope with environmental changes in temperature, either warming or cooling. For example, specific behaviors, such as vertical migration, can also help to reduce or avoid thermal stress. However, for copepods inhabiting coastal and semi-enclosed waters, such as *P. grani*, positive thermal anomalies can occur tens of meters down the water column, making exposure to thermal stress unavoidable. Many copepod species produce diapause eggs to overcome the unfavorable period (Marcus, 1996). *P. grani* is typically considered a thermophile species and in nature may rely on the production of resting eggs to survive the winter season (Guerrero and Rodríguez, 1998). In this regard, we did not observe morphologically distinct eggs that could be categorized as diapause eggs throughout all the egg counts out under the inverted microscope.

Our results show that the warm-reared copepod population may have the advantage to tolerate heat spikes at short time scales; nevertheless, under prolonged thermal stress situations like those under heatwave events (Smith *et al*., 2023), the differential advantage of the warm population still exists but diminish in magnitude. Our study also highlights the importance to include other key variables, besides survival, to assess the vulnerability of a species to thermal stress. Thus, the warm-reared copepods exhibited a much higher recruitment potential due to the combined effects on survival and fecundity. Further life-history traits should also be taken into account for a better comprehension of the thermal response of a species in nature.

Although we did not explore the viability of subsequent offspring at the test temperatures, some studies suggest that early stages may exhibit more plasticity than previously thought (Tangwancharoen and Burton, 2014; Klockmann *et al*., 2017; Holmes-Hackerd *et al*., 2023), and that temperature at development can improve the tolerance of adult stages (van Heerwaarden *et al*., 2016). Over long timescales, trans- or intergenerational mechanisms can emerge and provide further resilience to the organisms, increasing their chances to overcome extreme events. However, the evidences in this regard are equivocal. For instance, Geerts *et al*. (2015) reported that *Daphnia* recovered from a burial in an egg bank for over 40 years had lower thermal tolerance than some collected recently, subjected to current warming trends. Similarly, other studies show examples of recent local adaptation in heat tolerance in ectotherms (Brans *et al*., 2017; Diamond *et al*., 2017). Nevertheless, there is mounting evidence of the presence of physiological limits to heat tolerance that could also reduce the future acclimation capacity of species to extreme events (Hoffmann *et al*., 2013; Morgan *et al*., 2020; van Heerwaarden and Kellermann, 2020; Sasaki and Dam, 2021a). The long-term changes in heat tolerance and vital rates in response will have a significant influence on the local persistence of a species, as well as the re-organization of marine assemblages.

## CONCLUSIONS

Long-term warming has been found to enhance tolerance to acute extreme temperatures in marine ectotherms. However, it is crucial to consider the duration of the stress and the choice of the metric used, as they can significantly affect our assessment of a species’ thermal tolerance. When the calanoid copepod *P. grani* was reared for multiple generations at elevated temperatures, it exhibited increased survival and reproduction rates at the upper thermal range. This response enhances the species’ potential to persist during extreme heat events. While establishing and maintaining marine cultures and conducting long-term laboratory experiments with marine invertebrates can be challenging, the available data provides valuable insights that can contribute to our understanding of field conditions and help forecast ecological trends.

## Supplementary Material

Suplementary_JPR-2023-034_fbad037

## Data Availability

The dataset generated for this study is accessible through the Digital.CSIC repository https://doi.org/10.20350/digitalCSIC/15494.
