# Supplementary material for: Shifts in survival and reproduction after chronic warming enhance the potential of a marine copepod to persist under extreme heat events
Source: J Plankton Res. 2023 Sep 6;45(5):751–62. doi: 10.1093/plankt/fbad037 (PMC10539201; doi:10.1093/plankt/fbad037)
Supplement: Suplementary_JPR-2023-034_fbad037 [file suplementary_jpr-2023-034_fbad037.docx]

**Supplementary material for**

Shifts in survival and reproduction after chronic warming enhance the potential of a marine copepod to persist under extreme heat events

Carlos de Juan^1^, Albert Calbet^1^, Enric Saiz^1^

^1^Institut de Ciències del Mar (ICM), CSIC

Pg. Marítim de la Barceloneta 37-49, 08003 Barcelona, Spain

*Corresponding author: C. de Juan ([carlosdj@icm.csic.es](mailto:carlosdj@icm.csic.es))

ORCID ID:

C. de Juan: 0000-0002-1083-4418

A. Calbet: 0000-0003-1069-212X

E. Saiz: 0000-0003-2611-0067


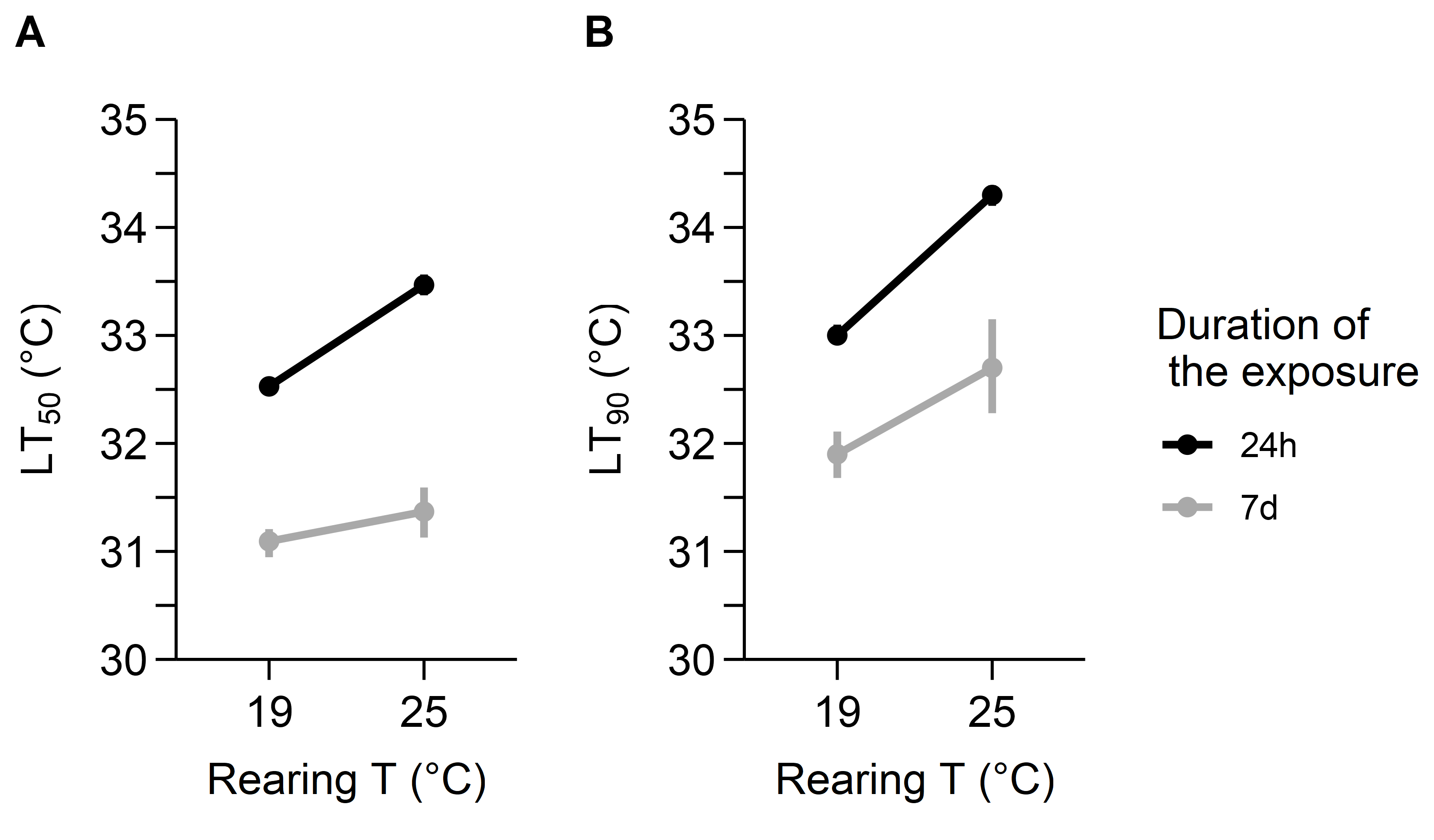


**Fig. S1** (A) LT_50_ and (B) LT_90_ after 24 hours (black) and 7 days (grey) exposure to elevated temperatures of the *P. grani* females reared during >18 generations at control (19°C) and warm (25°C) conditions.

**Fig. S2** (A) Survival, (B) egg production rate, and (C) potential recruitment after 7 days of exposure to extreme temperatures of *P. grani* reared at control (19°C) and warm conditions (25°C) during >18 generations. Potential recruitment was obtained by multiplying the survival rate after 7 days per the egg production rate at each rearing temperature. Each point shows the average of three replicates and standard error. Lines show linear regression of the descending phase of the thermal performance curve. p-value indicates significant differences between slopes.
